# Retrospective and Systematic Analysis of Causes and Outcomes of Thrombotic Microangiopathies in Routine Clinical Practice: An 11-Year Study

**DOI:** 10.3389/fmed.2021.566678

**Published:** 2021-02-26

**Authors:** Nicolas Henry, Chloé Mellaza, Nicolas Fage, François Beloncle, Franck Genevieve, Guillaume Legendre, Corentin Orvain, Anne-Sophie Garnier, Maud Cousin, Virginie Besson, Jean-François Subra, Agnès Duveau, Jean-François Augusto, Benoit Brilland

**Affiliations:** ^1^Service de Néphrologie-Dialyse-Transplantation, Université d'Angers, Centre Hospitalier Universitaire (CHU) Angers, Angers, France; ^2^Service de Médecine Intensive et Réanimation, Médecine Hyperbare, Université d'Angers, Centre Hospitalier Universitaire (CHU) Angers, Angers, France; ^3^Laboratoire d'Hématologie, Université d'Angers, Centre Hospitalier Universitaire (CHU) Angers, Angers, France; ^4^Département de Gynécologie et Obstétrique, Université d'Angers, Centre Hospitalier Universitaire (CHU) Angers, Angers, France; ^5^Service d'Hématologie, Université d'Angers, Centre Hospitalier Universitaire (CHU) Angers, Angers, France

**Keywords:** Thrombotic microangiopathies, etiology, primary, secondary, thrombotic thrombocytopenic purpura, hemolytic uremic syndrome, real-life

## Abstract

**Background:** Thrombotic microangiopathies (TMAs) are highly suspected in patients showing mechanical hemolytic anemia, thrombocytopenia, and haptoglobin consumption. Primary [thrombotic thrombocytopenic purpura (TTP) and atypical hemolytic uremic syndrome] and secondary TMA are considered. Even if ADAMTS13 measurements and alternative complement pathway explorations have greatly improved the ability to identify primary TMA, their diagnosis remains difficult, and their frequency relative to that of secondary TMA is undetermined. The objectives of the present study were, therefore, to describe the etiologies, management, and the outcomes of patients presenting with TMA in real-life clinical practice.

**Methods:** We conducted a retrospective study between 01/01/2008 and 31/12/2018 that included all consecutive patients presenting with biological TMA syndrome at admission or developing during hospitalization. Patients were identified from the laboratory databases, and their medical files were reviewed to confirm TMA diagnosis, to determine etiology, and to analyze their therapeutic management and outcomes.

**Results:** During this period, 239 patients with a full TMA biological syndrome were identified, and the TMA diagnosis was finally confirmed in 216 (90.4%) after the cases were reviewed. Primary TMAs (thrombotic thrombocytopenic purpura or atypical hemolytic uremic syndrome) were diagnosed in 20 of 216 patients (9.3%). Typical HUS was diagnosed in eight patients (3.7%), and the most frequent secondary TMAs were HELLP syndrome (79/216, 36.6%) and active malignancies (30/219, 13.9%). ADAMTS13 measurements and alternative complement pathway analyses were performed in a minority of patients. Multiple factors identified as TMA triggers were present in most patients, in 55% of patients with primary TMA, vs. 44.7% of patients with secondary TMA (*p* = 0.377). Death occurred in 57 patients (23.4%) during follow-up, and dialysis was required in 51 patients (23.6%). Active malignancies [odds ratio (OR) 13.7], transplantation (OR 4.43), male sex (OR 2.89), and older age (OR 1.07) were significantly associated with death.

**Conclusion:** Secondary TMAs represent many TMA causes in patients presenting a full TMA biological syndrome during routine clinical practice. Multiple factors favoring TMA are present in about half of primary or secondary TMA. ADAMTS13 and complement pathway were poorly explored in our cohort. The risk of death is particularly high in patients with malignancies as compared with patients with other TMA.

## Introduction

Thrombotic microangiopathies (TMAs) are defined by the presence of thrombi in small arterioles and capillaries (1). Thrombosis affects microcirculation and leads to tissue ischemia and organ failure (2). Although the confirmation of TMA diagnosis relies on histological features, a biopsy of an affected organ is rarely performed. Not only do TMAs have, in most cases, a characteristic biological presentation, but also biopsy is often contraindicated, given the bleeding risk. Mechanical hemolytic anemia, schistocytosis, and thrombocytopenia are strongly suggestive of TMA. As in other hemolytic anemia syndromes, haptoglobin consumption, elevated LDH, and elevated free bilirubin levels are also detected.

The understanding of biological mechanisms implicated in TMA development has greatly improved over the past three decades, allowing for the individualization of entities with specific pathophysiology (3, 4). In parallel with the pathophysiological understanding of TMA, the classification of TMA has been enriched with new entities and has further enabled the identification of several favoring or precipitating factors (1, 5). The latter can act as triggers of TMA development in patients with conditions that make them pre-disposed to primary TMA, i.e., genetic defects, or can induce TMA by themselves. TMAs are usually classified into two subsets, primary and secondary. Primary TMAs include thrombotic thrombocytopenic purpura (TTP) and atypical hemolytic uremic syndrome (aHUS). TTP is associated with low or undetectable ADAMTS13 activity (usually <10%), which may be related to a rare genetic defect in the ADAMTS13 gene (Upshaw–Schulman syndrome) or is, in most cases, an autoimmune disease associated with auto-antibodies neutralizing the enzymatic functions of ADAMTS13 (6). aHUS is related to several inherited or acquired abnormalities affecting complement alternative pathway (cAP) (7) that result in its permanent activation. Primary TMA can manifest at all ages and frequently develops after the occurrence of triggers that induce endothelial injury. Secondary TMAs, however, include numerous conditions or diseases that have been associated with TMA development. In secondary TMA, genetic defects and autoimmune abnormalities are rarely detected, and the microvascular endothelial injuries are driven by other factors altogether. The pathophysiology of secondary forms is, therefore, less well-defined, and as a result, diagnosis is rendered much more difficult. In the light of this, several classifications have been proposed to guide clinicians to the right diagnosis. Among secondary TMAs, typical HUS (tHUS) induced by the endothelial toxicity of Shiga toxins from *Escherichia coli* is the most well-defined secondary TMA. However, numerous other conditions such as a solid organ or bone marrow transplantation, drugs, pregnancy (HELLP syndrome), malignant hypertension, and malignancies can be associated with TMA development (1, 8–11). In such cases, the mechanisms of TMA are largely unknown and frequently multifactorial, and the therapeutic management of these patients is not well-codified.

The treatment of primary TMA has greatly evolved in the last few years using specific drugs such as rituximab in TTP and eculizumab in aHUS (12–15). In these conditions, mortality and organ lesions (mainly brain and kidney injuries) occur early, within the first days or weeks. A patient's prognosis is very dependent upon any delays to commencing with possible specific treatment. It is, therefore, crucial to identify these patients among the flood of those with a secondary cause.

The relative frequency of primary to secondary TMA has been poorly analyzed in the literature (16). Moreover, the management of patients with secondary TMA and their prognosis in comparison with patients with primary TMA are not well-known.

In the present study, we identified all consecutive patients with biological features of TMA (full biological TMA syndrome) in a period spanning 11 years. The objective of the study was to analyze the relative frequency of primary and secondary TMA, their presentation, and their therapeutic management. We also analyzed the prognosis of patients according to the causes of their TMA.

## Methods

### Selection of Patients

Patients admitted to the University Hospital of Angers between 01/01/2008 and 31/12/2018 and presenting with a full biological TMA syndrome were included in the study. The concomitant association of anemia defined full biological TMA syndrome (<12 g/dl in females and 13 g/dl in males), with thrombopenia ≤ 150 G/L, schistocytosis ≥0.5%, and a decreased haptoglobin level ≤0.4 g/L. These patients were identified from the database of our hematological laboratory. Two datasets listing patients with (1) thrombocytopenia and schistocytosis screening and (2) haptoglobin measurements during the study were extracted and merged. Adult patients (>18 years old) with concomitant thrombocytopenia, schistocytosis, and a decreased haptoglobin level were included in a systematic review of medical records after anemia had been confirmed. The study protocol complied with the standards of the Ethics Committee of the Angers University Hospital (no. 2019/12).

### Identification and Classification of Thrombotic Microangiopathic Patients

Medical records of patients identified with a full TMA biological syndrome were analyzed by four physicians (NH, CM, BB, and JFA) trained in nephrology, hematology, and critical care medicine, to confirm or rule out the diagnosis. In patients with a TMA diagnosis, its cause was determined after a hierarchical analysis according to current classifications. In a first step, patients with ADAMTS13 <10% were classified as having TTP. Next, patients with Shiga toxin-positive bacteria (detected in stool culture or polymerase chain reaction) were classified as having tHUS. The other following TMA causes were systematically considered: patients with HELLP syndrome, drugs known to induce TMA, graft vs. host disease (GvHD)-associated TMA, cancers, autoimmune diseases, malignant hypertension, and infections (excluding those related to Shiga toxin-producing bacteria). Patients with TMA and acute renal failure but without evidence of other secondary TMA causes and/or with cAP abnormalities known to be associated with TMA were classified as having aHUS. Patients without evidence of any of the causes of secondary TMA listed earlier, with rare secondary causes of TMA and without evidence of cAP abnormalities, were classified in “other secondary TMA causes.” Patients with multiple TMA potential causes, for whom clinical file review could not determine a predominant mechanism, were also classified in “other secondary TMA causes” (Supplementary Table 1).

In some patients with primary or secondary TMA, several TMA causes could be present. In patients with primary TMA, other possible causes were considered as favoring factors. Patients with secondary TMA were classified within the most probable diagnosis after medical file review, and other associated conditions were considered as favoring factors. Factors considered to favor TMA were concomitant infection, pregnancy, past or present history of malignancy, drugs known to induce TMA, transplantation, autoimmune diseases, and B12 deficiency.

### Therapeutic Management and Outcomes

Plasma exchange and/or plasma infusion, eculizumab and rituximab use, and the requirement for transfusion or dialysis were identified in medical charts and collected. Outcomes, including death or need for renal replacement therapy, were also collected.

### Statistical Analysis

Continuous variables are presented as median (minimum–maximum). Categorical variables are presented as the absolute value and percentage. Differences between groups were analyzed using the χ^2^ test (or Fisher exact test if necessary) for categorical variables and the Mann–Whitney U test for continuous variables. Logistic univariate analysis was used to examine factors associated with the outcomes. *P*-value < 0.05 were considered significant. Statistical analysis was performed using SPSS software® 23.0 and Graphpad Prism®.

## Results

### Baseline Characteristics

**Figure 1:**
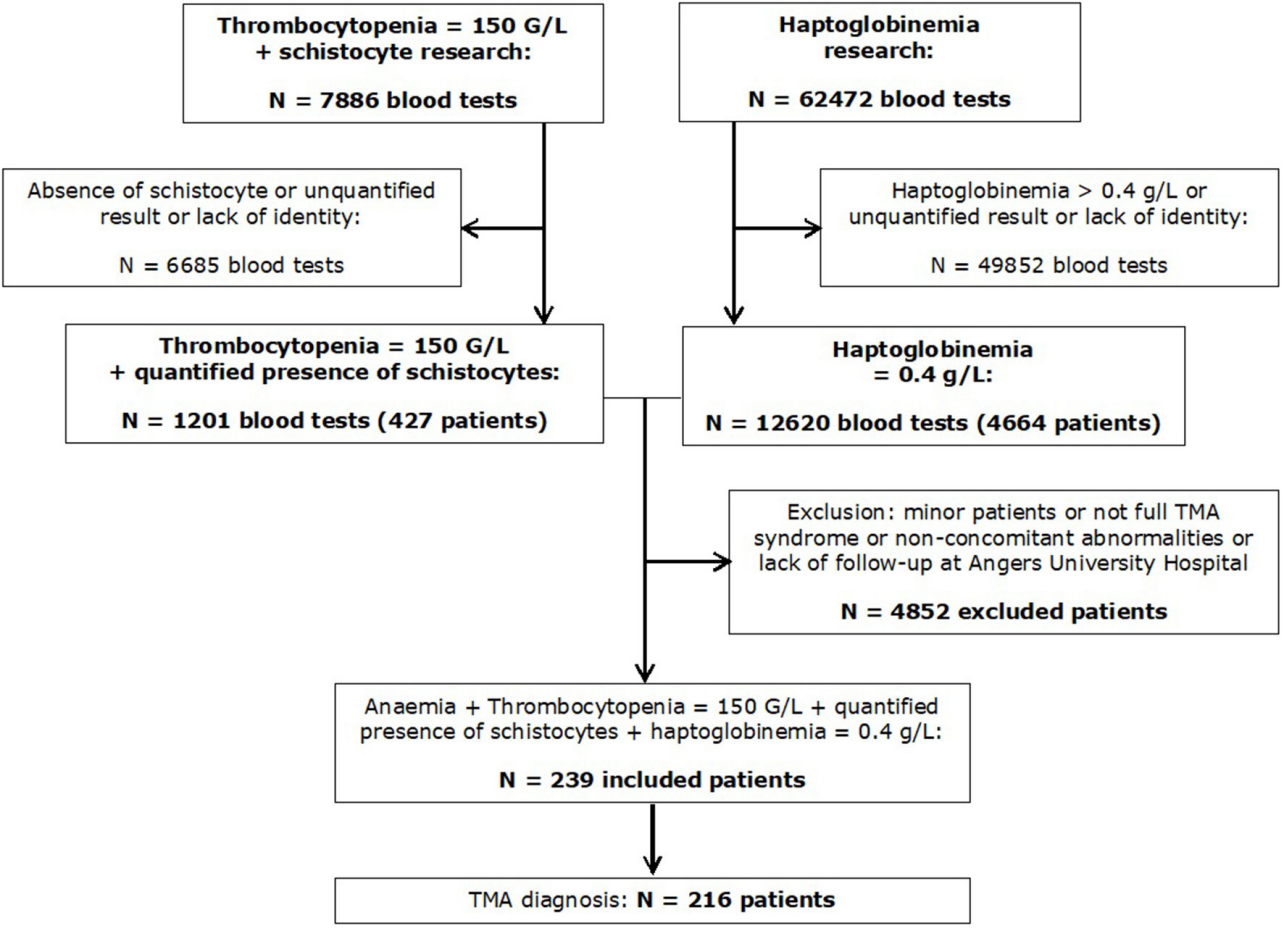
Flowchart of the study.

During the period mentioned earlier, we identified 427 patients with thrombocytopenia and schistocytosis ≥0.5% and 4,664 patients with haptoglobin ≤0.4 g/L. After cross-referencing the datasets, we could identify 239 patients with a full biological TMA syndrome. After the review of medical charts, 216 patients were finally diagnosed with TMA, whereas 23 had no evidence of TMA (Figure 1). The diagnosis retained in these 23 patients after reviewing their medical files are given in Supplementary Table 2. Patients with TMA were initially admitted to 17 different medical and surgical departments (Supplementary Figure 1). Among the 216 patients, 81 (37.5%) were admitted to a critical care unit at least partially during their hospitalization.

TMA patients were predominantly females (74.1%), with a median age of 45.3 ± 18.5 years. Hypertension and chronic kidney disease (estimated glomerular filtration rate <60 ml/min/1.73 m^2^) were found in 54 (25%) and 21 (9.7%) patients, respectively. Primary TMA (TTP or aHUS) was diagnosed in 20 patients (9.3%), with mostly acquired TTP (11 patients, 5.1%), whereas secondary TMA represented 90.7% of all TMA cases. Among secondary TMA, HELLP syndrome and active cancer-associated TMA were the most frequent diagnosis, in 36.6 and 13.9%, respectively. In the case of the latter, solid cancer featured in 19 patients and acute haemopathy in 11 patients. Other secondary TMA causes were less frequently observed, with tHUS representing 3.7% (Table 1 and Supplementary Figure 2). A subgroup of 30 patients was classified as “other TMA.” Some of these patients had a rare TMA cause or had no identified cause of TMA or multiple causes of TMA, not allowing their classification within other groups. The characteristics of patients classified in this subgroup of TMA are given in Supplementary Table 3. Surprisingly, TMA diagnosis was not mentioned within the medical file at the end of the initial admission for 53 of 216 (24.4%) patients. These patients were classified as having mainly secondary TMA after file review (cancer, *n* = 18; aGVHD, *n* = 12; undetermined TMA cause, *n* = 11). ADAMTS13 determination was performed in only 25% of patients, and, therefore, the diagnosis of TTP may have been missed in some patients. C3 and C4 complement fraction concentration and alternative complement pathway were studied in 33.8 and 19.4% of the patients of the cohort, respectively (Supplementary Table 4).

**Table 1:** Clinical and biological presentation of TMA according to etiology.

**Characteristics**	**Primary TMA**		**Secondary TMA**								
***N* (%)**	***n*** **=** **20 (9.3)**		***n*** **=** **196 (90.7)**								
	**TTP**	**aHUS**	**tHUS**	**HELLP syndrome**	**Malignant HBP**	**Malignancies**	**Drugs**	**Infections**	**AID**	**aGVHD**	**Others**
	***n* = 12 (5.6)**	***n* = 8 (3.7)**	***n* = 8 (3.7)**	***n* = 79 (36.6)**	***n* = 12 (5.6)**	***n* = 30 (13.9)**	***n* = 11 (5.1)**	***n* = 5 (2.3)**	***n* = 5 (2.3)**	***n* = 16 (7.4)**	***n* = 30 (13.9)**
**Baseline characteristics**											
Age (years)	59.2 (31–83)	36.9 (24–79)	63.9 (34–83)	29.8 (20–45)	35.6 (22–75)	63.6 (21–86)	59.1 (27–74)	63.8 (49–83)	72.3 (45–85)	53.4 (24–70)	39.5 (23–93)
Females (%)	7 (58.3)	6 (75.0)	6 (75.0)	79 (100)	5 (41.7)	20 (66.6)	5 (45.4)	3 (60.0)	2 (40.0)	8 (50.0)	17 (56.7)
Neurological signs	8 (66.6)	6 (75.0)	7 (87.5)	25 (31.6)	7 (58.3)	17 (56.7)	2 (18.2)	2 (40.0)	0 (0)	5 (31.2)	8 (26.7)
Diarrhea	3 (25.0)	0 (0)	8 (100)	0 (0)	0 (0)	0 (0)	1 (9.1)	0 (0)	0 (0)	9 (56.2)	4 (13.3)
AKI	9 (75.0)	8 (100)	8 (100)	26 (32.9)	10 (83.3)	15 (50.0)	7 (63.6)	5 (100)	5 (100)	8 (50.0)	14 (46.7)
**Biological presentation**											
Hemoglobin, g/Dl	7.3 (5.5–9.7)	7.5 (4.5–10.9)	9.5 (5.8–11.5)	9.8 (5.6–13)	7.2 (5.6–10.4)	7.6 (5.2–10.7)	6.9 (5.7–11.3)	6.1 (3.4–13.0)	8.0 (6.4–10.6)	8.5 (6.4–10.5)	9.6 (4.6–13.0)
Platelet count, G/L	10 (5–75)	68 (24–106)	42 (23–111)	38 (9–145)	97 (39–108)	48 (4–131)	53 (8–143)	41 (9–72)	94 (59–124)	15 (10–41)	78 (7–136)
LDH, UI/L	847 (618–1,852)	1,569 (278–4,200)	1,365 (412–3,709)	1,982 (276–6,760)	935 (297–1,955)	1,700 (288–6,546)	558 (221–1,648)	526 (478–4880)	992 (510–2318)	489 (150–14,540)	412 (240–5,900)
Schizocytes, *n* (%)											
0.5–1%	2 (16.7)	2 (25.0)	0 (0)	31 (39.2)	5 (41.7)	4 (13.3)	5 (45.5)	0 (0)	1 (20.0)	7 (43.8)	15 (50.0)
1–3%	1 (8.4)	3 (37.5)	4 (50.0)	40 (50.7)	4 (33.3)	11 (36.7)	3 (27.3)	3 (60.0)	3 (60.0)	5 (31.3)	8 (26.7)
3–5%	2 (16.7)	1 (12.5)	2 (25.0)	5 (6.3)	1 (8.3)	7 (23.3)	3 (27.3)	0 (0)	1 (20.0)	2 (12.5)	2 (6.7)
5–10%	5 (41.6)	2 (25.0)	2 (25.0)	2 (2.5)	1 (8.3)	7 (23.3)	1 (9.1)	2 (40.0)	0 (0)	2 (12.5)	2 (6.7)
>10%	2 (16.7)	0(0)	0 (0)	1 (1.3)	1 (8.3)	1 (3.3)	0 (0)	0 (0)	0 (0)	0 (0)	1 (3.3)
Elevated free bilirubin, *n* (%)	7 (58.3)	3 (37.5)	6 (75.0)	47 (59.5)	3 (25.0)	19 (63.3)	5 (45.5)	4 (80.0)	0 (0)	10 (62.5)	14 (46.7)
Elevated LE, *n* (%)	5 (41.7)	1 (12.5)	6 (75.0)	79 (100)	3 (25.0)	8 (26.7)	4 (36.4)	5 (100)	5 (100)	9 (56.3)	12 (40.0)
Fibrinogen, g/L	4.2 (1.5–7.8)	4.8 (4.3–9.9)	3.4 (2.6–8.3)	3.9 (0.3–8.4)	3.8 (2.5–5.9)	2.4 (0.3–7.5)	3.0 (2.8–4.7)	2.9 (1.7–5.0)	4.0 (2.4–6.7)	3.1 (1.8–11.5)	3.4 (2.3–8.8)
Prothrombin time (%)	78.0 (76–83)	55.5 (24–80)	90.0 (66–113)	97.0 (77–109)	96.0 (61–117)	73.0 (32–106)	88.0 (57–103)	56.0 (21–86)	60.5 (59–62)	89.0 (19–112)	79.5 (19–106)
CRP, mg/L	6 (3–105)	27 (4–65)	19.5 (3–45)	28 (4–111)	4 (3–52)	50 (4–347)	29 (3–98)	9 (3–322)	122.5 (76–169)	29.5 (3–488)	31.6 (3–230)
Serum creatinine, μmol/L	94 (62–1,131)	795 (300–1,631)	268 (97–844)	81.5 (33–746)	1,087 (120–1,491)	245 (30–700)	258 (63–499)	244 (138–350)	287 (106–505)	100 (50–343)	383 (64–962)
Proteinuria, g/L	1.0 (0.2–5.2)	4.4 (0.4–9.8)	1.8 (1.5–4.5)	7.6 (0.0–31.5)	2.7 (0.6–8)	1.1 (0.0–24.0)	1.1 (0.27.1)	0.2 (0.1–1.1)	0.4 (0.2–0.5)	0.6 (0.4–3.5)	1.8 (0.1–24)
Albuminemia, g/L	36 (14–49)	28 (21–42)	32 (23–34)	22 (18–36)	37 (21–45)	26 (17–43)	30 (24–43)	32 (31–33)	29 (21–40)	29 (19–36)	26 (20–38)

### Clinical and Biological Presentation According to Thrombotic Microangiopathy Cause

Table 1 reports the clinical and biological presentation according to the TMA etiology. Patients with aHUS tended to be younger compared with patients with TTP. In secondary TMA, patients with HELLP syndrome were younger than those with malignancies. Neurological signs were present at admission with variable frequencies according to TMA causes. They were present in most patients with primary TMA, either TTP or aHUS. Neurological symptoms exhibited greater variety in secondary TMA, most frequently observed in patients with malignant hypertension and malignancies. Acute kidney injury was present in all patients with aHUS and tHUS, in most patients with TTP, and with a variable frequency in other secondary TMA causes. Serum creatinine was greatly higher in patients with aHUS and malignant hypertension, as compared with other causes of TMA. The level of proteinuria showed a similar trend. However, although 75% of TTP patients developed acute kidney injury, their median serum creatinine level at admission was 94 μmol/L and therefore in the same range as HELLP syndrome patients. Diarrhea was present in all patients with tHUS and in most patients with aGvHD but was very inconstantly observed in other conditions. Anemia was present in all patients but with variable severity. Thrombocytopenia was particularly profound in patients with TTP.

### Analysis of Thrombotic Microangiopathy-Favoring Factors

Except for patients with HELLP syndrome and active autoimmune diseases, the presence of an additional factor known to favor TMA was frequent (Table 2). There was no difference in the frequency of patients with at least one additional factor between patients with primary TMA and patients with secondary TMA (55.0 vs. 44.7%, *p* = 0.377). The most frequent additional factor was a history of malignancy and concomitant administration of a drug known to favor TMA, mostly gemcitabine or calcineurin inhibitor. Patients with aGvHD and drug-associated TMA tended to have more associated factors than patients with primary TMA and with other secondary causes (Table 2).

**Table 2:** TMA-favoring factors according to TMA cause.

**Characteristics**	**Primary**		**Secondary**								
	**TTP**	**aHUS**	**tHUS**	**HELLP syndrome**	**Malignant HBP**	**Malignancies**	**Drugs**	**Infections**	**AID**	**aGVHD**	**Others**
	***n* = 12**	***n* = 8**	***n* = 8**	***n* = 79**	***n* = 12**	***n* = 30**	***n* = 11**	***n* = 5**	***n* = 5**	***n* = 16**	***n* = 30**
**Infections**	**0**	**0**	**8**	**0**	**0**	**8**	**0**	**5**	**0**	**12**	**1**
Shiga toxin *E. coli*	0	0	8	0	0	0	0	0	0	0	0
Bacteria	0	0	0	0	0	2	0	3	0	4	0
Virus	0	0	0	0	0	2	0	1	0	10	0
Fungus	0	0	0	0	0	2	0	1	0	5	1
Undetermined	0	0	0	0	0	2	0	1	0	0	0
**Pregnancy**	**1**	**2**	**0**	**79**	**0**	**0**	**0**	**0**	**0**	**0**	**11**
**History of Malignancy**	**2**	**1**	**3**	**1**	**2**	**26**	**9**	**3**	**0**	**13**	**5**
Solid cancer	0	1	3	0	2	18	6	2	0	1	2
Hematological malignancy	0	0	0	0	0	9	3	1	0	13	3
**Actual Malignancy**	**0**	**2**	**0**	**0**	**0**	**30**	**0**	**0**	**0**	**0**	**0**
Solid cancer	0	1	0	0	0	19	0	0	0	0	0
Hematological malignancy	0	1	0	0	0	11	0	0	0	0	0
**Drugs**	**5**	**1**	**0**	**0**	**0**	**7**	**11**	**0**	**0**	**12**	**1**
**Transplantation**	**3**	**1**	**0**	**0**	**0**	**2**	**3**	**1**	**0**	**16**	**7**
Solid organ transplantation	2	1	0	0	0	0	1	0	0	0	4
Stem cell transplantation	1	0	0	0	0	2	2	1	0	16	3
**Autoimmune disease**	**0**	**0**	**0**	0	**1**	**0**	**0**	**0**	**5**	**0**	**0**
**B12 deficiency**	**0**	**0**	**0**	**0**	**0**	**0**	**0**	**0**	**0**	**0**	**3**
**No identified factor**	0	0	0	0	0	0	0	0	0	0	**7**
Mean number of other factors per patient^*^	**0.92**	**0.87**	**0.37**	**0.01**	**0.25**	**1.43**	**1.09**	**0.8**	**0**	**2.93**	**-**

* *When the factor was considered as the main etiology of TMA, it was not counted as a favoring factor*.

### Management of Thrombotic Microangiopathy

Red blood cell transfusion was used in 43.5% of patients and platelet transfusion in 22.7% of patients. Plasma exchange or plasma infusion was initiated in 28.2% of patients. Rituximab and eculizumab were administered in 7.9 and 3.7% of patients, respectively. As expected, plasma exchange or plasma infusion was used in most patients with primary TMA. Rituximab was administered in 50% of patients with TTP and at a lower frequency in other TMA groups. Eculizumab was used in eight patients, five of them with aHUS or tHUS (Table 3).

**Table 3:** Therapeutic management of TMA patients.

**Treatment, *n* (%)**		**Primary TMA**		**Secondary TMA**								
	**All**	**TTP**	**aHUS**	**tHUS**	**HELLP syndrome**	**Malignant HBP**	**Malignancies**	**Drugs**	**Infections**	**AID**	**aGVHD**	**Others**
	***n* = 216**	***n* = 12**	***n* = 8**	***n* = 8**	***n* = 79**	***n* = 12**	***n* = 30**	***n* = 11**	***n* = 5**	***n* = 5**	***n* = 16**	***n* = 30**
Plasma exchange	40 (18.5)	9 (75.0)	6 (75.0)	6 (75.0)	0 (0)	1 (8.3)	5 (16.6)	3 (27.3)	2 (40.0)	3 (60.0)	1 (6.25)	4 (13.3)
Plasma infusion	30 (13.9)	2 (16.6)	2 (25.0)	2 (25.0)	10 (12.7)	0 (0)	3 (10.0)	3 (27.3)	0 (0)	0 (0)	2 (12.5)	6 (20.0)
PE or PI	61 (28.2)	9 (75.0)	6 (75.0)	6 (75.0)	10 (12.7)	1 (8.3)	6 (20.0)	6 (54.5)	2 (40.0)	3 (60.0)	3 (18.8)	9 (30.0)
Eculizumab	8 (3.7)	1 (8.3)	3 (37.5)	2 (20.0)	0 (0)	0 (0)	1 (3.3)	1 (9.1)	0 (0)	0 (0)	0 (0)	0 (0)
Rituximab	17 (7.9)	6 (50.0)	1 (12.5)	0 (0)	0 (0)	0 (0)	3 (10.0)	0 (0)	0 (0)	2 (40.0)	4 (25.0)	1 (3.3)
Red blood cell transfusion	94 (43.5)	8 (66.7)	4 (50.0)	4 (50.0)	14 (17.7)	6 (50.0)	19 (63.3)	5 (45.4)	2 (40.0)	4 (80.0)	15 (93.8)	13 (43.3)
Platelet transfusion	49 (22.7)	3 (25.0)	1 (12.5)	1 (12.5)	7 (8.9)	1 (8.3)	12 (40.0)	5 (45.4)	0 (0)	0 (0)	14 (87.5)	5 (16.6)
Dialysis (during first admission)	51 (23.6)	3 (25.0)	6 (75.0)	3 (37.5)	6 (7.59)	10 (83.3)	5 (16.7)	3 (27.3)	3 (60.0)	2 (40.0)	2 (12.5)	8 (26.7)

### Outcomes and Factors Associated With Prognosis

Fifty-one (23.6%) patients required dialysis during initial admission, and 57 (23.4%) died during follow-up at a median time of 42 days (1–1,796) from initial admission. Using univariate analysis, older age [odds ratio (OR) 1.07], male sex (OR 2.89), active malignancies (OR 13.7), and transplantation (OR 4.43) were associated with an increased risk of death. Primary TMA (OR 3.00), aHUS, and tHUS (OR 4.84) were associated with dialysis initiation during the first admission, whereas male sex (OR 0.49) and HELLP syndrome (OR 0.17) were associated with a decreased risk of requiring dialysis (Table 4).

**Table 4:** Univariate analysis of factors associated with outcomes.

	N^*^	**Death (n** **=** **57)**		N^*^	**RRT (n** **=** **51)**	
		**OR (95% CI)**	***P***		**OR (95% CI)**	***P***
**Patient characteristics**						
Age^**^	-	1.07 (1.05–1.09)	** <0.001**	-	1.02 (0.98–1.02)	0.823
Male (yes)	32	2.89 (1.50–5.55)	**0.001**	19	0.49 (0.25–0.96)	**0.037**
**TMA etiology**						
Primary TMA (vs. secondary)	7	1.57 (0.59–4.16)	0.362	9	3.00 (1.17–7.71)	**0.023**
TTP (yes)	5	2.09 (0.63–6.86)	0.225	3	1.08 (0.28–4.16)	0.907
aHUS or tHUS (vs. others)	3	0.62 (0.17–2.27)	0.475	9	4.84 (1.70–13.7)	**0.003**
HELLP syndrome (vs. others)	0	-		6	0.17 (0.07–0.42)	** <0.001**
Active malignancies (vs. others)	24	13.7 (5.67–33.3)	** <0.001**	7	0.89 (0.36–2.20)	0.802
Transplantation (vs. others)	18	4.43 (2.05–9.58)	** <0.001**	10	1.51 (0.66–3.42)	0.328
aGvHD (vs. others)	10	5.42 (1.87–15.7)	**0.002**	2	0.44 (0.01–2.00)	0.289
**Need for RRT** (vs. no RRT)	51	1.22 (0.61–2.45)	0.576	-	-	-

* *Number of events in subgroup*.

** *per year*.

## Discussion

The present study gives an overview of TMA etiology and management in routine clinical practice. The major finding of this work is that secondary TMAs are largely much more frequent than primary TMAs (TTP and aHUS). Importantly, using our biologically based selection and systematic medical file review, we were able to conclude TMA diagnosis in ~25% of patients for whom the diagnosis was not clearly mentioned within the medical file. These patients had secondary TMA related to various causes. This observation probably reflects the complexity of TMA diagnosis, especially when multiple factors are involved. Data from TMA registries show a higher proportion of primary TMA. As an example, in the Oklahoma TTP-HUS registry, TTP represented 15% of TMA between 1999 and 2007 (17) and 43% in the UK TTP registry between 2009 and 2013 (18). These discrepancies are probably explained because entries into these registers are linked to plasma exchange requirement and by ADAMTS13 measurement, respectively. Thus, they include selected populations and do not, in fact, reflect the frequency of TMA, nor their distribution in “real-life” conditions. Rather, in the present study, we undertook an unbiased analysis of all consecutive patients with full TMA biological syndrome. Interestingly, a very recent study with a methodology like that used in the present study showed similar frequencies of primary and secondary TMA (16): TTP and aHUS represented 5.9% of TMA, slightly below the 9.3% observed in our study.

It is important to note that ADAMTS13 measurement and cAP analysis were performed in only a minority of patients in our cohort. Therefore, some patients may have been misclassified as having a secondary TMA. This would be in line with recent research showing abnormalities in alternative complement pathway proteins, notably patients with clinical features of malignant hypertension (19, 20) or with TMA associated with drugs, such as calcineurin inhibitors used in transplant patients (21). More recently, patients with HELLP syndrome have also been shown to present a higher incidence of germline alternative complement pathway gene mutations (22). The explanation for such a low rate of patients with specific investigations in our study is likely to be multifaceted, reflecting an unawareness of these relatively new data, the rarity of TMA, and the variable TMA experience of the clinician.

We observed the presence of favorable/precipitating factors that can act as “triggers” in ~50% of patients. Interestingly, their frequency showed no significant difference between patients with primary and secondary TMAs. Therefore, this observation supports the concept of the “multi-hit” theory of TMA (23). TMA has been shown to occur in ~10% of patients with aGvHD (24). It is interesting to note that it was the condition with the higher number of concomitant triggers in our study.

Finally, primary TMAs, for which treatment has been better codified in past years, represent a minority of TMA cases. HELLP syndrome and active malignancies were the most highly represented causes of secondary TMA in our cohort. The repartition between secondary causes in our study is closely aligned with those observed in the study of Bayer et al. (16). However, we observed far fewer patients with infection-associated TMA (2.3%). These discrepancies are probably linked to different methods for selecting patients and for classifying them between the two studies. First, in Bayer et al.'s study, screening constituted a combination of automatized medical file analysis and the inclusion of patients with partial biological TMA syndrome. The differences in patient selection probably explain why Bayer et al. included many more patients. Second, in our study, we considered infectious events as triggers rather than as causative agents *per se*.

In line with previous data, the mortality rate was high in our study, and death occurred early after TMA diagnosis. In the univariate analysis, active malignancies, transplantation, and aGvHD were significantly associated with a higher risk of death. These observations are in line with the clinical experience we have of TMA and with the few available reports on these topics (24–26). Notably, the differentiation between chemotherapy-induced TMA and cancer-associated TMA represents a challenge, especially when medical files are reviewed retrospectively. Moreover, these patients do not usually undergo extensive biological explorations given their very limited prognosis; this in turn also limits what can be achieved through reviewing files. We may, therefore, have misclassified some of them.

In conclusion, our study highlights that more than 90% of patients with full TMA biological syndrome have secondary TMAs, which are associated with very high mortality. We also point out that it is not uncommon for the diagnosis of TMA not to be mentioned, especially in patients diagnosed with secondary TMA. Therefore, these results suggest that more attention and clinical research should focus on secondary TMA to understand better the mechanisms implicated in it, especially with systematic cAP and ADAMTS13 exploration to rule out primary TMA, improve their classification and codify their treatment.

## Data Availability Statement

The datasets presented in this article are not readily available because The datasets generated for this study are available on request to the corresponding author. Requests to access the datasets should be directed to jfaugusto@chu-angers.fr.

## Ethics Statement

The studies involving human participants were reviewed and approved by Ethics Committee of the Angers University Hospital (n°2019/12). Written informed consent for participation was not required for this study in accordance with the national legislation and the institutional requirements.

## Author Contributions

NH, CM, and J-FA contributed to the conception and design of the study. J-FA supervised the project. NH and CM organized the database and wrote the first draft of the manuscript. NH, CM, J-FA and BB performed the statistical analysis. J-FA and BB provided critical revision of the manuscript. All authors participated in patients care. All authors read and approved the submitted version.

## Conflict of Interest

The authors declare that the research was conducted in the absence of any commercial or financial relationships that could be construed as a potential conflict of interest.

## Supplementary Material

Supplementary Figure 1 Site of initial hospital admission.

Supplementary Figure 2 Number of patients according to TMA etiology.

Supplementary Table 1 Diagnosis criteria for classifying the causes of TMA.

Supplementary Table 2 Patients with full TMA biological syndrome without TMA.

Supplementary Table 3 Characteristics of patients with “other” TMA causes.

Supplementary Table 4 ADAMTS13, complement and autoimmune evaluation.
