# Supplementary material for: Retrospective and Systematic Analysis of Causes and Outcomes of Thrombotic Microangiopathies in Routine Clinical Practice: An 11-Year Study
Source: Front Med (Lausanne). 2021 Feb 26;8:566678. doi: 10.3389/fmed.2021.566678 (PMC7952313; doi:10.3389/fmed.2021.566678)
Supplement: Supplementary Figure 1 — Site of initial hospital admission. [file Table_1.docx]

# SUPPLEMENTARY MATERIAL

**Supplementary Table 1. Diagnosis criteria for classifying the causes of TMA.**

**Supplementary Table 2. Patients with full TMA biological syndrome without TMA.**

**Supplementary Table 3. Characteristics of patients with “other” TMA causes.**

**Supplementary Table 4. ADAMTS13, complement and autoimmune evaluation.**

**Supplementary Figure 1. Site of initial hospital admission.**

**Supplementary Figure 2. Number of patients according to TMA aetiology.**

## Supplementary Table 1. Diagnosis criteria for classifying the causes of TMA.

| **TMA aetiology** | **Diagnosis criteria** |
| --- | --- |
| TTP | TMA diagnosis with ADAMTS13 < 10% and/or anti-ADAMTS13 antibody positivity |
| aHUS | TMA and acute renal failure without evidence of other secondary TMA causes and/or with complement alternative pathways abnormalities known to be associated with TMA |
| tHUS | TMA with Shiga toxin-positive bacteria detected in stool culture or PCR |
| HELLP syndrome | TMA in pregnant women with haemolysis, thrombopenia and elevated liver enzymes |
| Malignant HBP | TMA associated with hypertensive retinopathy and diastolic blood pressure > 120 mmHg |
| Malignancies | TMA associated with newly diagnosed cancer or active carcinologic disease |
| Drugs | TMA occurring in patients undergoing treatment with drugs known to induce TMA |
| Infections | TMA associated with infection, but with no evidence of *E.coli* Shiga toxin detection and without any other cause of TMA (i.e., drug-induced TMA) |
| AID | TMA developing in patients with active autoimmune disease (e.g. SLE) and without other causes of TMA |
| aGVHD | TMA developing in patients with aGVHD |
| Others | TMA without evidence of any of the causes of secondary TMA listed above, with rare secondary causes of TMA and without evidence of complement alternative pathway abnormality |

## Supplementary Table 2. Patients with full TMA biological syndrome without TMA.

| **Patient** | **Gender** | **Age (years)** | **Hb (g/dL)** | **Platelets (G/L)** | **Schistocytosis (%)** | **Haptoglobin (g/L)** | **Medical diagnosis** | **RRT** | **Death** |
| --- | --- | --- | --- | --- | --- | --- | --- | --- | --- |
| 1 | M | 79 | 7.6 | 57 | 1-3% | <0.3 | DIC, pulmonary infection | no | yes |
| 2 | M | 75 | 7.2 | 44 | 3-5% | <0.3 | Q fever, haemolytic auto-immune anaemia | no | no |
| 3 | M | 30 | 10.5 | 118 | 0.5-1% | <0.3 | Cirrhosis, transjugular intrahepatic shunt | no | yes |
| 4 | M | 68 | 6.9 | 18 | 0.5-1% | <0.3 | Pancytopenia, schistocytosis once | yes | yes |
| 5 | F | 25 | 7.3 | 25 | 0.5-1% | <0.3 | ECMO for ARDS | yes | yes |
| 6 | F | 56 | 6.7 | 76 | 1-3% | <0.3 | Kidney graft haematoma | yes | no |
| 7 | F | 65 | 7.6 | 68 | 0.5-1% | <0.3 | Child Pugh C cirrhosis, schistocytosis once | no | no |
| 8 | F | 81 | 9.0 | 15 | 0.5-1% | <0.3 | Cholangiocarcinoma, DIC, MAS | no | yes |
| 9 | F | 47 | 5.2 | 63 | 0.5-1% | <0.3 | Autoimmune haemolytic anaemia, cirrhosis, schistocytosis once | no | no |
| 10 | M | 29 | 6.5 | 48 | 0.5-1% | <0.3 | Pancytopenia, dialysis membrane reaction | yes | no |
| 11 | F | 63 | 6.3 | 74 | 1-3% | <0.3 | Haemorrhagic shock, haemolysis after massive RBC transfusion | no | no |
| 12 | M | 58 | 9.8 | 139 | 5-10% | <0.3 | Mechanical heart valve haemolytic anaemia | no | no |
| 13 | M | 32 | 3.6 | 100 | 0.5-1% | <0.3 | Autoimmune haemolytic anaemia, myelomonocytic leukaemia | no | no |
| 14 | F | 83 | 8.7 | 133 | 0.5-1% | <0.3 | Mechanical heart valve haemolytic anaemia | no | yes |
| 15 | M | 31 | 7.2 | 1 | 0.5-1% | <0.3 | Epstein-Barr virus-associated MAS | no | no |
| 16 | F | 76 | 9.5 | 139 | 0.5-1% | 0.3 | Cardiogenic shock, schistocytosis once | no | yes |
| 17 | M | 51 | 7.5 | 50 | 1-3% | <0.3 | Cardiogenic shock, MOD | yes | no |
| 18 | F | 70 | 7.5 | 30 | 0.5-1% | <0.3 | ITP, TAVR | no | no |
| 19 | F | 59 | 6.1 | 131 | 0.5-1% | <0.3 | Autoimmune haemolytic anaemia, | no | no |
| 20 | F | 79 | 7.8 | 24 | 0.5-1% | 0.4 | DIC, aortic valve replacement, HIT | no | no |
| 21 | F | 56 | 7.2 | 24 | 3-5% | <0.3 | Mechanical heart valve haemolytic anaemia | yes | yes |
| 22 | F | 61 | 10.6 | 59 | 1-3% | <0.3 | ITP | no | no |
| 23 | F | 39 | 6.4 | 147 | 0.5-1% | <0.3 | Cirrhosis | no | no |

ARDS, Acute respiratory distress syndrome; DIC, diffuse intravascular coagulation; ECMO, extracorporeal membrane oxygenation; HIT, heparin-induced thrombocytopenia; ITP, idiopathic thrombocytopenic purpura; MAS, macrophage activation syndrome; MOD, multi-visceral organ dysfunction; RBC, red blood cell; TAVR, transcatheter aortic valve replacement.

## Supplementary Table 3. Characteristics of patients with “other” TMA causes.

| **Patient** | **Gender**  **(M/F)** | **Age (years)** | **Hb (g/dL)** | **Platelets (G/L)** | **Schistocytes**  **(%)** | **Haptoglobin (g/L)** | **Dialysis**  **(Y/N)** | **Pregnancy**  **(Y/N)** | **Cancer**  **(Y/N)** | **Infection**  **(Y/N)** | **AID**  **(Y/N)** | **Death**  **(Y/N)** | **Medical diagnosis** |
| --- | --- | --- | --- | --- | --- | --- | --- | --- | --- | --- | --- | --- | --- |
| 1 = 4 | F | 33 | 10 | 69 | 1-3% | 0.3 | Y | Y | N | N | N | N | **Pregnancy without HELLP syndrome** **or PTT or aHUS** |
| 2 = 11 | M | 75 | 6.1 | 31 | 3-5% | <0.2 | N | N | Y | N | Y | N | **TMA of unknown aetiology**  History of colorectal cancer and heparin-induced thrombocytopenia |
| 3 = 14 | M | 76 | 9.2 | 78 | 1-3% | <0.2 | Y | N | N | N | N | Y | **TMA of unknown aetiology**  History of MGUS, deceased of unknown cause |
| 4 = 16 | F | 77 | 9.6 | 99 | 1-3% | <0.2 | N | N | N | N | N | N | **TMA of unknown aetiology** |
| 5 = 20 | M | 64 | **12.6** | 9 | 1-3% | <0.2 | y | N | N | N | N | Y | **TMA of unknown aetiology**, death by multi-visceral organ dysfunction |
| 6 = 26 | M | 52 | 6.5 | 57 | 3-5% | <0.2 | N | N | Y | N | Y | Y | **TMA of unknown aetiology**, death by TMA  History of Waldenström disease and anti-Mag neuropathy |
| 7 = 30 | M | 23 | 10 | 21 | >10% | <0.2 | N | N | N | Y | N | N | **Disseminated intravascular coagulation** and probable sepsis of unknown origin. TMA of unknown aetiology |
| 8 = 31 | F | 41 | 12 | 12 | 5-10% | <0.2 | Y | N | N | N | N | N | **TMA of unknown aetiology**  History of stem cell transplantation idiopathic aplastic anaemia, no PTT/SHUa |
| 9 = 49 | F | 57 | 10.7 | 63 | 1-3% | <0.2 | N | N | Y | Y | N | Y | **Septic shock, history of stem cell transplantation for myelofibrosis** |
| 10 = 72 | F | 51 | 9.5 | 7 | 0.5-1% | <0.2 | N | N | Y | N | N | N | **TMA of unknown aetiology** |
| 11 = 93 | F | 26 | 6.5 | 49 | 0.5-1% | <0.2 | N | N | N | N | N | N | **B12 deficiency** |
| 12 = 110 | F | 27 | 6.9 | 62 | 0.5-1% | <0.2 | N | Y | N | N | N | N | **Pregnancy without HELLP syndrome**, no PTT/aHUS research  **+ disseminated intravascular coagulation** |
| 13 = 115 | F | 31 | 10 | 110 | 0.5-1% | 0.3 | N | Y | N | N | N | N | **Pregnancy without HELLP syndrome**, no PTT/aHUS research |
| 14 = 118 | F | 33 | 10.7 | 117 | 0.5-1% | <0.2 | N | Y | N | N | N | N | **Pregnancy without HELLP syndrome**, no PTT/aHUS research |
| 15 = 119 | F | 26 | 10.1 | 131 | 1-3% | <0.2 | N | Y | N | N | N | N | **Pregnancy without HELLP syndrome**, no PTT/aHUS research |
| 16 = 123 | F | 37 | 6.5 | 40 | 0.5-1% | <0.2 | N | Y | N | N | N | N | **Pregnancy without HELLP syndrome**, no PTT/aHUS research  **+ disseminated intravascular coagulation** |
| 17 = 136 | M | 24 | 6.3 | 71 | 0.5-1% | <0.2 | Y | N | N | N | N | N | **Acute renal graft rejection + anticalcineurin** |
| 18 = 138 | F | 32 | 4.8 | 61 | 0.5-1% | 0.3 | N | Y | N | Y | N | N | **Pregnancy without HELLP syndrome**, no PTT/aHUS research  History of B viral hepatitis |
| 19 = 141 | M | 30 | 7.1 | 88 | 1-3% | <0.2 | Y | N | N | Y | Y | N | **Acute renal graft rejection** **+ anticalcineurin + Cryptococcosis**  History of lupus |
| 20 = 160 | F | 28 | 9.5 | 130 | 0.5-1% | <0.2 | Y | N | Y | N | N | N | **Acute renal graft rejection + anticalcineurin,**  History of Hodgkin disease |
| 21 = 163 | M | 53 | 6.6 | 32 | 5-10% | <0.2 | Y | N | N | N | N | N | **Disseminated intravascular coagulation** (no follow up)  History of renal graft (no rejection) + anticalcineurin |
| 22 = 171 | F | 37 | 9.9 | 89 | 0.5-1% | <0.2 | N | Y | N | N | N | N | **Pregnancy without HELLP syndrome**, no PTT/aHUS research |
| 23 = 177 | M | 93 | 8 | 62 | 0.5-1% | <0.2 | N | N | Y | N | Y | N | **TMA of unknown aetiology**  History of colorectal cancer, Waldenström disease and psoriasis |
| 24 = 183 | M | 50 | 7.2 | 32 | 0.5-1% | 0.2 | N | N | N | N | N | N | **TMA of unknown aetiology** |
| 25 = 189 | M | 42 | 6.3 | 86 | 0.5-1% | <0.2 | N | N | N | N | N | N | **B12 deficiency** |
| 26 = 192 | F | 19 | 6.8 | 121 | 0.5-1% | <0.2 | N | Y | N | N | N | N | **Pregnancy without HELLP syndrome**, no PTT/aHUS research |
| 27 = 194 | F | 27 | 10 | 82 | 0.5-1% | <0.2 | N | Y | N | N | N | N | **Pregnancy without HELLP syndrome**, no PTT/aHUS research |
| 28 = 208 | F | 33 | 12.4 | 136 | 0.5-1% | <0.2 | N | Y | N | N | N | N | **Pregnancy without HELLP syndrome**, no PTT/aHUS research |
| 29 = 210 | F | 79 | 5.9 | 61 | 0.5-1% | <0.2 | N | N | N | N | Y | N | **B12 deficiency, history of thyroiditis** |
| 30 = 237 | F | 57 | 4.6 | 115 | 1-3% | <0.2 | N | N | N | N | N | N | **TMA of unknown aetiology** |

## Supplementary Table 4. ADAMTS13, complement and autoimmune evaluation.

|  | **All, n=216** |
| --- | --- |
|  |  |
| **ADAMTS13 determination, n (%)** | 54 (25) |
| ADAMTS13 activity < 10%* | 11 (20.4) |
| Anti-ADAMT13 antibody* | 10 (18.5) |
| **Complement analysis, n (%)** | - |
| C3 and C4 complement determination | 73 (33.8) |
| Low C3 concentration* | 2 (2.74) |
| Low C4 concentration* | 1 (1.4) |
| Alternative complement pathway analysis | 42 (19.4) |
| Abnormal result* | 9 (21.4) |
| CFH mutation | 4 (44.4) |
| MCP mutation | 4 (44.4) |
| Anti-FH antibody | 1 (11.2) |
| **Antinuclear antibodies, n (%)** | 49 (22.7) |
| Positivity (> 1/100)* | 15 (30.6) |
| **Anti-ds DNA antibodies** | 49 (22.7) |
| Positivity* | 2 (13.3) |

* Amongst those tested

## Supplementary Figure 1. Site of initial hospital admission.

**
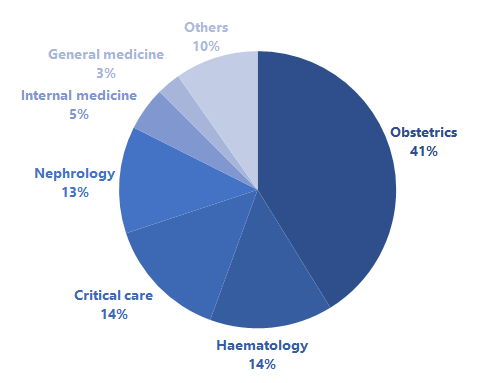
**

## Supplementary Figure 2. Number of patients according to TMA aetiology.
